# The Effects of Reward on Associative Memory Depend on Unitization Depths

**DOI:** 10.3389/fpsyg.2022.839144

**Published:** 2022-02-14

**Authors:** Chunping Yan, Qianqian Ding, Meng Wu, Jinfu Zhu

**Affiliations:** School of Psychology, Xinxiang Medical University, Xinxiang, China

**Keywords:** associative memory, reward effect, relational retrieval, unitization, ERP

## Abstract

Previous studies have found that reward effect is stronger for more difficult to retrieve items, but whether this effect holds true for the associative memory remains unclear too. We investigated the effects and neural mechanisms of the different unitization depths and reward sets on encoding associative memory using event-related potentials (ERPs), which were recorded through a Neuroscan system with a 64-channel electrode cap according to the international 10–20 system, and five electrodes (Fz, FCz, Cz, CPz, and Pz) were selected for analysis. Thirty healthy college students took part in this study. During encoding, participants were carried out two encoding tasks, a congruity-judgment task with high unitization and a color-judgment task with low unitization, with half of the items rewarded. The test phase was conducted immediately after the encoding phase. The results for false alarm rates and Prs (i.e., hit rates for old pairs minus false alarm rates for new pairs) in relational retrieval revealed that the reward differences in the color-judgment task were greater than those in the congruity-judgment task. The ERP results further showed significant reward effects (i.e., the reward significantly improved the average amplitudes compared to no reward) at P300 (300–500 ms) and LPP (500–800 ms) in the color-judgment task both for intact and rearranged items, and the reward effects at LPP (electrodes Fz, FCz, Cz, CPz, and Pz) were distributed more widely than the reward effects at P300 (electrodes Fz and FCz) in the color-judgment task. These results suggest that reward provided a greater boost when retrieving associative memory of low unitized items.

## Introduction

Episodic memory is the memory of past events (Tulving, 1985). Episodic memory includes both item memory and associative memory. Item memory is the memory of specific items, which involves item retrieval; associative memory is the memory of the relationship between two items or the item and its context, which involves relational retrieval. Binding is one of the main cognitive processing mechanisms involved in associative memory and is the process of combining different elements of memory into a whole (Cohen et al., 1999; Naveh-Benjamin, 2000). There are three types of associative memory: (1) intra-item associative memory, which is memory of items and their perceptual features (such as a word and its color); (2) within-domain inter-item associative memory, which is memory of two of the same type of elements independent of each other (such as a word and another word); and (3) between-domain inter-item associative memory, which is memory of different kinds of elements (such as a word and an image). Between-domain inter-item associative recognition requires more recollection support than within-domain inter-item associative recognition (Mayes et al., 2007). Familiarity and recollection represent two primary processes underlying memory recognition (Yonelinas, 2002). Dual-process theory distinguishes between familiarity and recollection: familiarity is the feeling of knowing about a learned item and is an automatic processing based on continuous changes in intensity; recollection is the extraction of the details learned item, which depends on attention resources (Yonelinas, 2002; Guez et al., 2019). Dual-process theory suggests that only recollection supports the relational retrieval of associative memory (Opitz and Cornell, 2006; Guez et al., 2019). In contrast, the unitization hypothesis holds that familiarity also supports associative memory retrieval when stimulus materials are unitized during encoding (Quamme et al., 2007; Bastin et al., 2013; Delhaye et al., 2017). People’s relational memory in daily life involves a wide range of fields, which often involves the binding of one kind of thing with another kind of thing. Therefore, associative memory merits further research. In this study, between-domain inter-item associative memory of different kinds of elements was explored.

Reward is often considered as a stimulus or thing desired that can be obtained by performing a specific task. Numerous studies have shown that monetary rewards can regulate attention and promote the processing of reward-related stimuli (Krebs et al., 2010; Veling and Aarts, 2010; Wei and Kang, 2014). Some studies have explored the effect of monetary reward on episodic memory, and they found that reward significantly improved recognition performance (Adcock et al., 2006; Halsband et al., 2012; Shigemune et al., 2014; Gruber et al., 2016; Yan et al., 2017; Chen and Wei, 2018). Of course, other researchers have explored whether the reward effect in memory is influenced by other factors. For example, Swirsky et al. (2020) explored the influence of processing level at encoding (gist vs. detail) and reward on memory retrieval and found that the accuracy (ACC) was higher in the gist condition (deeper, semantic levels of processing) than in the detail condition (shallower, perceptual levels of processing). Shigemune et al. (2017) also found that the reward effect on item recognition could be influenced by the difficulty level of retrieval. However, it is not clear whether the reward effect in associative memory differs at different processing depths.

Graf and Schacter (1989) proposed that unitization in associative memory is the process by which two or more previously separate items are integrated into a whole unit. Unitization can be divided into top-down unitization and bottom-up unitization depending on the direction of information flow (Tibon et al., 2014, 2017). According to Shao et al. (2016), top-down unitization relies on a set of instructions that process stimuli pairs into a single representation. Murray and Kensinger (2012) manipulated unitization using encoding strategies. In this study, we used two encoding tasks with varying unitization (high or low) in the encoding phase. We investigated the effects and neural mechanisms of encoding tasks with varying unitization depths and monetary rewards on between-domain inter-item associative memory, using event-related potential (ERP) measurements.

Related ERP studies on reward and memory have also shown significant reward effects (Eppinger et al., 2010; Yan et al., 2017). ERP studies have found that the P300 component (an early positive component that appears at approximately 300 ms poststimulus) and the LPP component (a late positive component) reflect physiological and psychological functions related to cognitive processes, such as perception and memory (Hada et al., 2000; Marini et al., 2011). ERP studies on reward memory have also shown significant reward effects in early P300 and late LPP (Eppinger et al., 2010; Marini et al., 2011; Halsband et al., 2012). P300 might reflect the initial attention allocation to the stimulus, whereas LPP might reflect the significance of the stimulus (Schupp et al., 2006; Foti and Hajcak, 2008; Hajcak et al., 2009). ERP studies have also shown that LPP might reflect memory encoding, as the average amplitudes of items with deeper processing strategies were more positive than those with shallower processing strategies (Guo et al., 2003; Marini et al., 2011). Previous ERP studies have shown that the average amplitudes of old images are more positive than those of new images, a phenomenon called the “old/new effect.” The FN400 old/new effect is an earlier frontal negative component that peaks at approximately 400 ms poststimulus and is associated with familiarity, while the LPC old/new effect is a late parietal positive component that peaks at approximately 600 ms poststimulus, and is associated with recollection (Curran and Hancock, 2007; Addante et al., 2012; Shayesteh et al., 2020).

This research explored the effects and neural mechanisms of the different unitization depths and reward on between-domain inter-item associative memory using ERPs, because ERPs have a high time resolution, which can illuminate the time course of the effects of different unitization depths and rewards during encoding and retrieval and better illustrate the differences between different levels of these factors over time. In this study, we introduced reward cues during encoding and instructed participants to carry out two encoding tasks: a congruity-judgment task and a color-judgment task. The congruity-judgment and color-judgment tasks, involving deep or shallow processing of the connection between words and images, respectively, corresponded to high and low unitization levels of associative memory. During retrieval, we utilized an associative recognition paradigm (Desaunay et al., 2020): participants were presented with some pairs of items that they had learned during encoding, called “intact” pairs; others that recombining two items they learned but did not encode at the same time, called “rearranged” pairs; and others made up of two completely novel pairs, called “new” pairs. Participants were asked to categorize the item pairs into “intact,” “rearranged,” or “new.” Based on previous studies, our hypothesis was that the reward differences at the Prs within the color-judgment task would be greater than those in the congruity-judgment task and that significant reward effects at P300 (early) or LPP (late) would only be found in the color-judgment tasks.

## Materials and Methods

### Participants

Thirty-three right-handed college students from Xinxiang Medical University took part in this study, and they were selected through the random sampling method from five grades’ undergraduates. The young population were selected based on a large number of relevant studies (Yan et al., 2017; Guez et al., 2019; Shayesteh et al., 2020). They had normal or corrected-to-normal vision and no history of neurological or psychiatric disorders. The study protocol was approved by the institutional ethics committee of Xinxiang Medical University in China, and all the methods were carried out in accordance with relevant guidelines. No vulnerable populations were involved in the present study. All participants provided signed informed consent prior to the experiment, conforming to the Declaration of Helsinki. To maintain a sufficient signal-to-noise ratio, the data from three participants were rejected due to the exclusion criteria of recording less than 16 trials under certain conditions. Therefore, the final analysis included data from 30 college students (mean age = 21.2 years; 15 of whom were male). At the end of the experiment, each participant received compensation.

### Materials

The target stimuli consisted of 240 color-neutral images selected from the Chinese Affective Picture System (Bai et al., 2005) and the International Affective Picture System (IAPS; Lang et al., 1993), which were uniform in size (433 × 310 pixels). In addition, 240 neutral Chinese two-character words were selected from the Modern Chinese frequency dictionary (Liu, 1990), which were uniform in size (250 × 128 pixels); half were red, and half were green. Twenty-two college students (12 males), who did not participate in the formal experiment, provided valence (1 = very unhappy, 9 = very cheerful) and arousal (1 = very calm, 9 = very excited) ratings for the images. The average valence and arousal scores of the images were 5.02 ± 0.52 and 4.05 ± 0.56, respectively. Those of the words were 4.99 ± 0.47 and 3.58 ± 0.45, respectively. Of these, 160 images and 160 words were used as study (old) items, and another 80 images and 80 words were used as test (new) items. All images and words were divided into four groups (congruity-judgment tasks under reward condition, congruity-judgment tasks under nonreward condition, color-judgment tasks under reward condition, and color-judgment tasks under nonreward condition); each group contained 60 images and 60 words (40 old items and 20 new items), and the four groups of images and words were matched on valence and arousal (see Table 1).

**Table 1:** The average valence and arousal ratings of the stimuli groups in this study.

	Stimuli	Group 1 (*n* = 60)	Group 2 (*n* = 60)	Group 3 (*n* = 60)	Group 4 (*n* = 60)	*F*_(3, 236)_	*p*
Valence	Picture	5.01 ± 0.06	5.00 ± 0.03	5.03 ± 0.05	4.99 ± 0.07	1.35	0.588
	Word	4.92 ± 0.04	5.05 ± 0.06	5.02 ± 0.05	5.00 ± 0.06	1.42	0.396
Arousal	Picture	4.08 ± 0.06	4.02 ± 0.05	4.03 ± 0.06	4.06 ± 0.06	1.18	0.317
	Word	3.82 ± 0.04	3.79 ± 0.06	3.71 ± 0.05	3.76 ± 0.04	1.25	0.226

The data after “±” in the table are the standard errors of the mean.

In the retrieval phase, these images and words were pseudorandomly combined to form image-word pairs, resulting in 320 image-word pairs (160 “intact,” 80 “rearranged,” and 80 “new”). We focused on the comparison between intact and rearranged pairs, demonstrated a relatively pure old/new effect of associative memory, based on previous studies on associative memory (Rhodes and Donaldson, 2008; Zheng et al., 2015; Han et al., 2018).

Ten neutral images were selected from IAPS, and 10 neutral words were selected from the Modern Chinese frequency dictionary as training materials; the training pictures and words did not appear in the formal experiment.

### Procedures

A 2 (encoding task: congruity-judgment task vs. color-judgment task) × 2 (reward type: reward vs. nonreward) within-subjects design was used.

The experimental procedure was compiled using the Presentation software (Neurobehavioral Systems, San Francisco, CA). Before the formal experiment, the participants were familiarized with the experimental process and keystroke responses through practice. Participants were told that they would complete congruity-judgment or color-judgment tasks involving images and words during encoding and then would complete the test phase after encoding. During encoding, the participants were told that they would obtain a monetary reward (RMB 0.20 for each object) if they correctly recognized and judged the rewarded items in the retrieval phase, while the nonrewarded items would provide a reward if they were correctly or wrongly judged, and a cumulative cash payment would be made at the end of the experiment.

**Figure 1:**
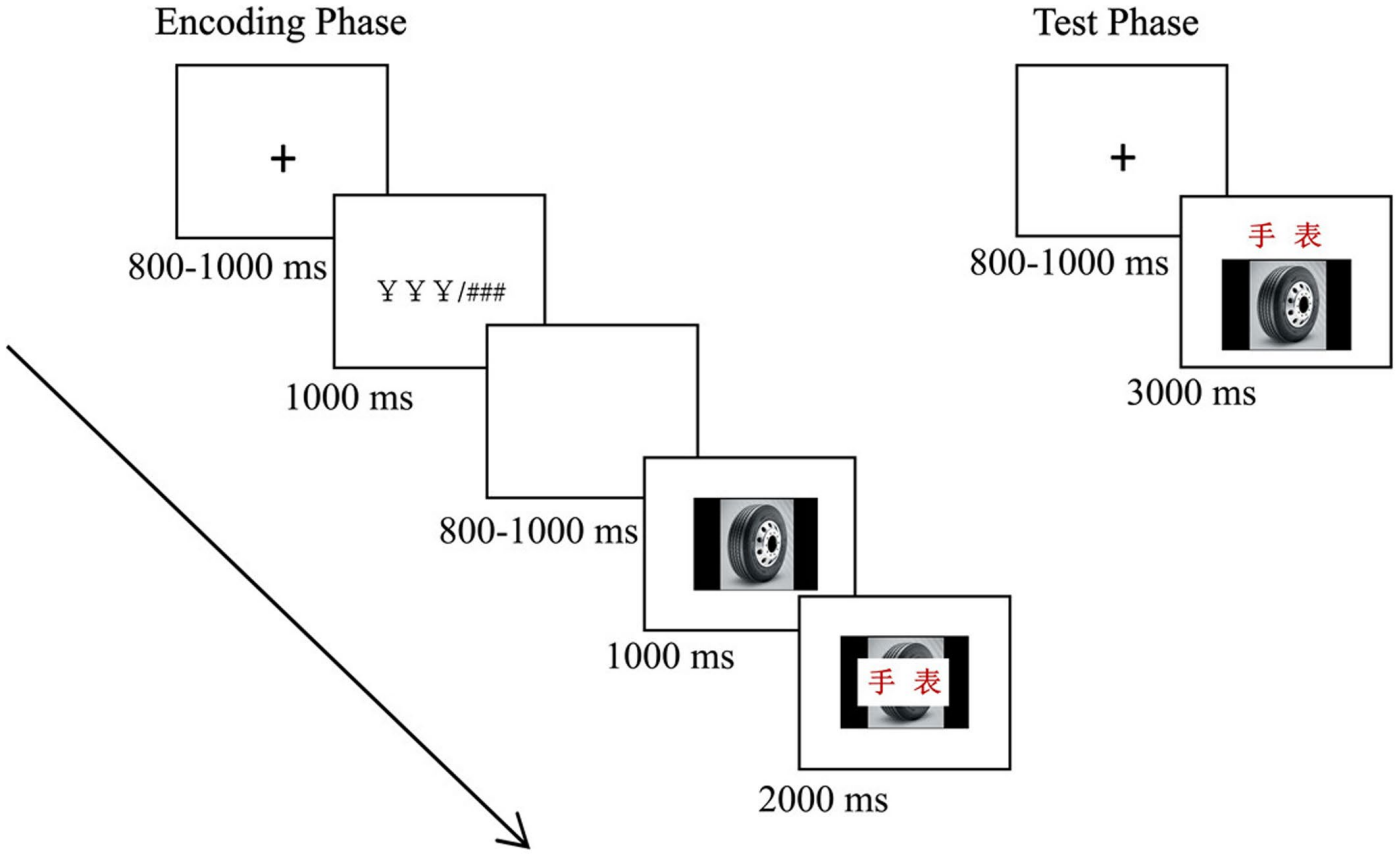
Schematic representations of a trial in the encoding phase (left) and test phase (right). See the text for details, and “手表” in the figure means “watch.”

The formal experiment included four blocks (two congruity-judgment tasks and two color-judgment tasks), each containing encoding and test phases (see Figure 1). In the encoding phase, the trials proceeded as follows: each trial began with a cross fixation point presented for a duration of 800–1,000 ms, followed by a reward cue (¥ ¥ ¥) or nonreward cue (# # #) for 1,000 ms; then, a blank screen was presented for 800–1,000 ms; after that, an image was presented for 1,000 ms; then, a Chinese two-character words was superimposed in the middle of the image; and they were presented together for 2000 ms. During this time, participants were asked to perform the corresponding task: the congruity-judgment task (determine whether the words matched the images) or the color-judgment task (judge the color of the word is green or red) by pressing “F” or “J” on the keyboard within the 2000 ms, and then the next trial began. In total, participants studied 40 image-word pairs in the encoding phase of each block. After encoding, participants were asked to complete a distraction task (i.e., repeatedly subtracting 3 from each number) for 1 min. After that, the test phase was carried out. Participants performed 60 trials, comprised of 20 trials of “intact” items, 20 trials of “rearranged” items, and 20 trials of “new” items. Each trial began with a cross fixation point present for 800–1,000 ms, followed by an image-word pair (words presented above the image) for 3,000 ms, and participants were asked to categorize the pair as “intact,” “rearranged,” or “new” by pressing “F,” “B,” or “J” on the keyboard within 3,000 ms (Han et al., 2018). Then, the next trial began. Participants were asked to make quick and accurate judgments. The order of the trials was pseudorandom and successive. All the reaction keys were counterbalanced between the left and right hands across the participants. The order of the four blocks was also counterbalanced across participants.

### ERP Recordings and Analysis

Brain electrical activity was recorded from 64 Ag-AgCl scalp sites according to the international 10–20 system in an elastic cap (Neuroscan Product). Electroencephalographic (EEG) was sampled online with a frequency of 500 Hz sampling rate with a 0.1–100 Hz bandpass filter. The reference electrode was placed on the left mastoid process, and the connection point was midway between FPz and Fz. An electrooculogram (EOG) air at the outer canthi of both eyes. Vertical EOGs were recorded with one pair of electrodes placed above and below the left eye, and horizontal EOGs were recorded with another pair at the outer canthi of both eyes. All electrodes were referenced online to the left mastoid and rereferenced offline to the average of the right and left mastoid recordings. EOG blink artifacts were corrected using a linear regression estimate (Hornberger et al., 2004; Picton et al., 2010; Wang et al., 2015). EEG/EOG signals (impedance <5 kΩ) were bandpass-filtered at 0.05–40 Hz, and the average amplitude 200 ms prior to stimulus (images in the encoding and image-word pairs in the test) onset was used for baseline correction. Trials with a voltage exceeding ±100 μV were excluded from the ERP analysis.

In this study, ERP data were used to analyze the EEG changes in two phases: the encoding and test phases. The time range of the EEG analysis was 0–2000 ms after image onset in the encoding phase and 0–1,000 ms after simultaneous word and image onset in the test phase. Referring to previous studies and based on the observation of amplitudes and topographic maps in this study, Hu et al. (2013) and Han et al. (2018) used similar experimental designs and reported such effects. Five representative midline electrodes were selected for analysis: frontal (Fz), frontocentral (FCz), central (Cz), centroparietal (CPz), and parietal (Pz) electrodes. Time windows (400–750 ms, 750–1,250 ms and 1,250–1850 ms) were selected in the encoding phase and in the test phase (300–500 ms and 500–800 ms) referring to Elliott et al. (2019), who used similar intervals. Our analyses focused on reward effects (P300 and LPP), encoding task effects (LPP), and their interaction with respect to “intact” items, as well as FN400 and LPC old/new (i.e., intact/rearranged) effects under each condition (Han et al., 2018).

To analyze the effect of the encoding task and reward type in both encoding and test phases, 2 (encoding task: congruity-judgment task and color-judgment task) × 2 (reward type: reward and nonreward) × 5 (electrode location: Fz, FCz, Cz, CPz, and Pz) repeated-measures ANOVAs were conducted on the average amplitudes of each time window. To analyze the old/new (i.e., intact/rearranged) effect, 2 (encoding task: congruity-judgment task and color-judgment task) × 2 (reward type: reward and nonreward) × 2 (item type: intact and rearranged) × 5 (electrode location: Fz, FCz, Cz, CPz, and Pz) repeated-measures ANOVAs were conducted on the average amplitudes of each time window. Repeated-measures ANOVAs were corrected using the Greenhouse–Geisser method (Greenhouse and Geisser, 1959). The alpha level was 0.05. Multiple comparisons or simple effect analyses were corrected using the Bonferroni correction. All data analyses were conducted using SPSS statistics software.

## Results

### Behavioral Data

The Shapiro–Wilk tests for normality suggested that the data under each condition were normally distributed (*Ws* > 0.92, *ps* > 0.05).

### Accuracy and Response Time in the Encoding Phase

Accuracy and response time (RT) were the dependent variables. A two-way repeated-measures ANOVAs on ACC and RT with the factors of encoding task (congruity-judgment task and color-judgment task) and reward type (reward and nonreward).

The ACC results did not find a significant main effect of encoding task [*F*_(1, 29)_ = 0.74, *p* = 0.396, $\eta_{p}^{2}$ = 0.02] or reward type [*F*_(1, 29)_ = 0.38, *p* = 0.540, $\eta_{p}^{2}$ = 0.01]; there was no interaction between the two factors [*F*_(1, 29)_ = 1.19, *p* = 0.148, $\eta_{p}^{2}$ = 0.03].

The RT results did not find a significant main effect of encoding task [*F*_(1, 29)_ = 2.68, *p* = 0.110, $\eta_{p}^{2}$ = 0.07] or reward type [*F*_(1, 29)_ = 1.57, *p* = 0.259, $\eta_{p}^{2}$ = 0.03], but there was a significant interaction between the two factors [*F*_(1, 29)_ = 4.57, *p* = 0.040, $\eta_{p}^{2}$ = 0.12]. Further simple effect analysis found that there was a response times were significantly longer in the congruity-judgment task (1839 ± 57 ms) than in the color-judgment task (1725 ± 55 ms, *p* = 0.026) under the reward condition, but there was no significant difference between the two under the nonreward condition (*p* = 0.392); rewarded items had shorter response times in the color-judgment task (*p* = 0.015) than nonrewarded items, but there was no significant difference between the two in the congruity-judgment task (*p* = 0.862).

### Behavioral Data in the Test Phase

To rule out the effect of the color of words (green or red) on recognition of these words, with ACC and response time (RT), respectively, as the dependent variable, a paired samples *T*-test was carried out with colors (green and red) as the factors. The results suggested that there was no significant difference in the ACC and RT between the green words and red words [*t*_(29)_ = −1.24, *p* = 0.312; *t*_(29)_ = 1.01, *p* = 0.546]. These indicated that the color of words (green or red) has no significant impact on the recognition of words.

### Prs in the Test Phase

**Figure 2:**
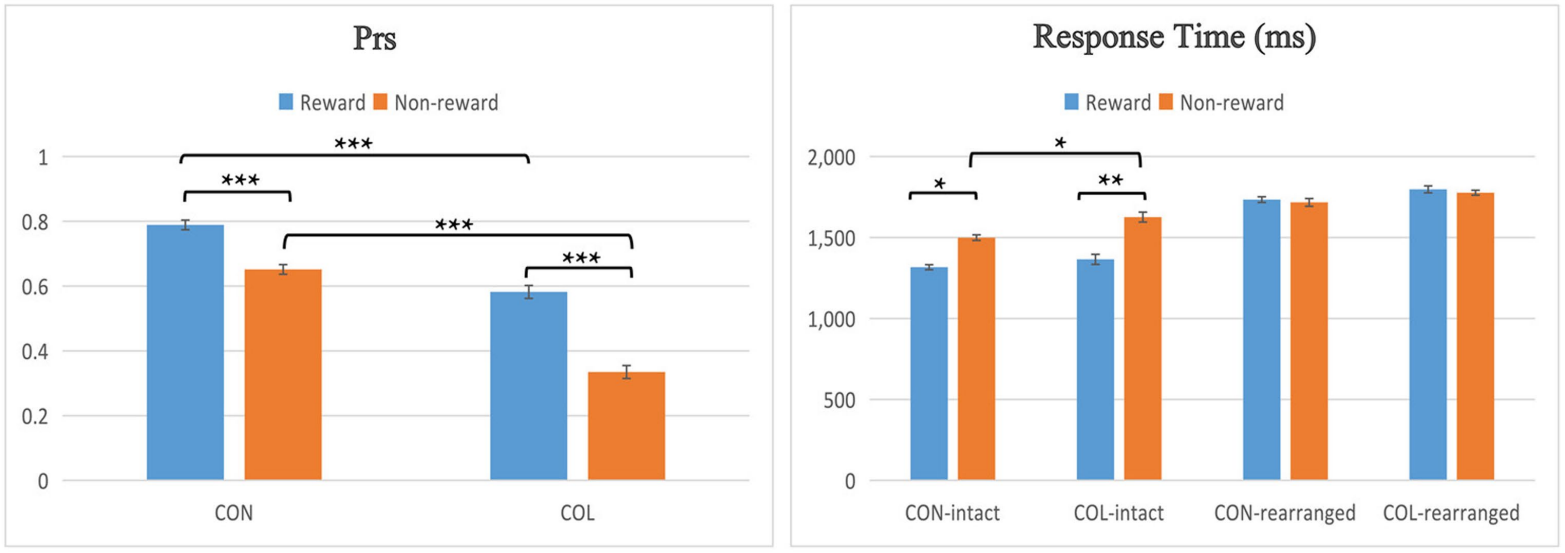
Behavioral performance across conditions in the test phase. Error bars represent standard errors of the mean, ^*^p < 0.05, ^**^p < 0.01, and ^***^p < 0.001.

Participants’ recognition performance is shown in Figure 2. False alarm rates for “rearranged” pairs were tested using a two-way repeated-measures ANOVA with the factors of encoding task (congruity-judgment task and color-judgment task) and reward type (reward and nonreward). The results showed a significant main effect of encoding task [*F*_(1, 29)_ = 42.79, *p* < 0.001, $\eta_{p}^{2}$ = 0.55]: the false alarm rate in the color-judgment task was significantly higher than that in the congruity-judgment task.

The discrimination ACC index Pr (the hit rate minus the false alarm rate) was used to determine memory ACC (Snodgrass and Corwin, 1988). In this study, the associative Pr was calculated as the hit rate for old/“intact” pairs minus the false alarm rate for “rearranged” pairs (Snodgrass and Corwin, 1988; Jäger et al., 2006; Han et al., 2018).

Prs were tested using a two-way repeated-measures ANOVA with the factors of encoding task (congruity-judgment task and color-judgment task) and reward type (reward and nonreward). There was a significant main effect of encoding task [*F*_(1, 29)_ = 118.00, *p* < 0.001, $\eta_{p}^{2}$ = 0.77], a significant main effect of reward type [*F*_(1, 29)_ = 43.48, *p* < 0.001, $\eta_{p}^{2}$ = 0.55], and a significant interaction between the two factors [*F*_(1, 29)_ = 7.24, *p* = 0.011, $\eta_{p}^{2}$ = 0.17]. Further simple effect analysis found that the Prs of the congruity-judgment task were significantly higher than those of the color-judgment task in either reward condition (*ps* < 0.001); the Prs of rewarded items were significantly higher than those of nonrewarded items in both tasks (*ps* < 0.001). In addition, because we observed that the reward differences of the color-judgment task may be greater than those of the congruity-judgment task, the reward differences (Prs of rewarded items minus those of nonrewarded items) between the congruity-judgment and color-judgment tasks were compared through a paired samples *t*-test, and the results indicated that the reward differences in the color-judgment task were significantly greater than in the congruity-judgment task [*t*_(31)_ = 3.04, *p* = 0.014].

### Response Time in the Test Phase

A three-way repeated-measures ANOVA on response times was performed with encoding task (congruity-judgment task and color-judgment task), item type (intact and rearranged), and reward type (reward and nonreward) as factors. There were no significant main effects of encoding task and reward type [*F*_(1, 29)_ = 1.10, *p* = 0.302, $\eta_{p}^{2}$ = 0.03; *F*_(1, 29)_ = 2.05, *p* = 0.161, $\eta_{p}^{2}$ = 0.06], but there was a significant main effect of response [*F*_(1, 29)_ = 119.99, *p* < 0.001, $\eta_{p}^{2}$ = 0.77] and a significant three-way interaction [*F*_(1, 29)_ = 7.06, *p* = 0.012, $\eta_{p}^{2}$ = 0.17]. Further simple effect analysis suggested that RTs in the congruity-judgment task were significantly shorter than the color-judgment task under nonreward condition for the old/intact items (*p* = 0.016); rewarded items had shorter response times than nonrewarded items both in the congruity-judgment and color-judgment tasks for the old/intact items (*p* = 0.015; *p* = 0.007); and the RTs were significantly shorter for the old/intact items than the rearranged items in both tasks (*ps* < 0.05).

### ERP Data

**Figure 3:**
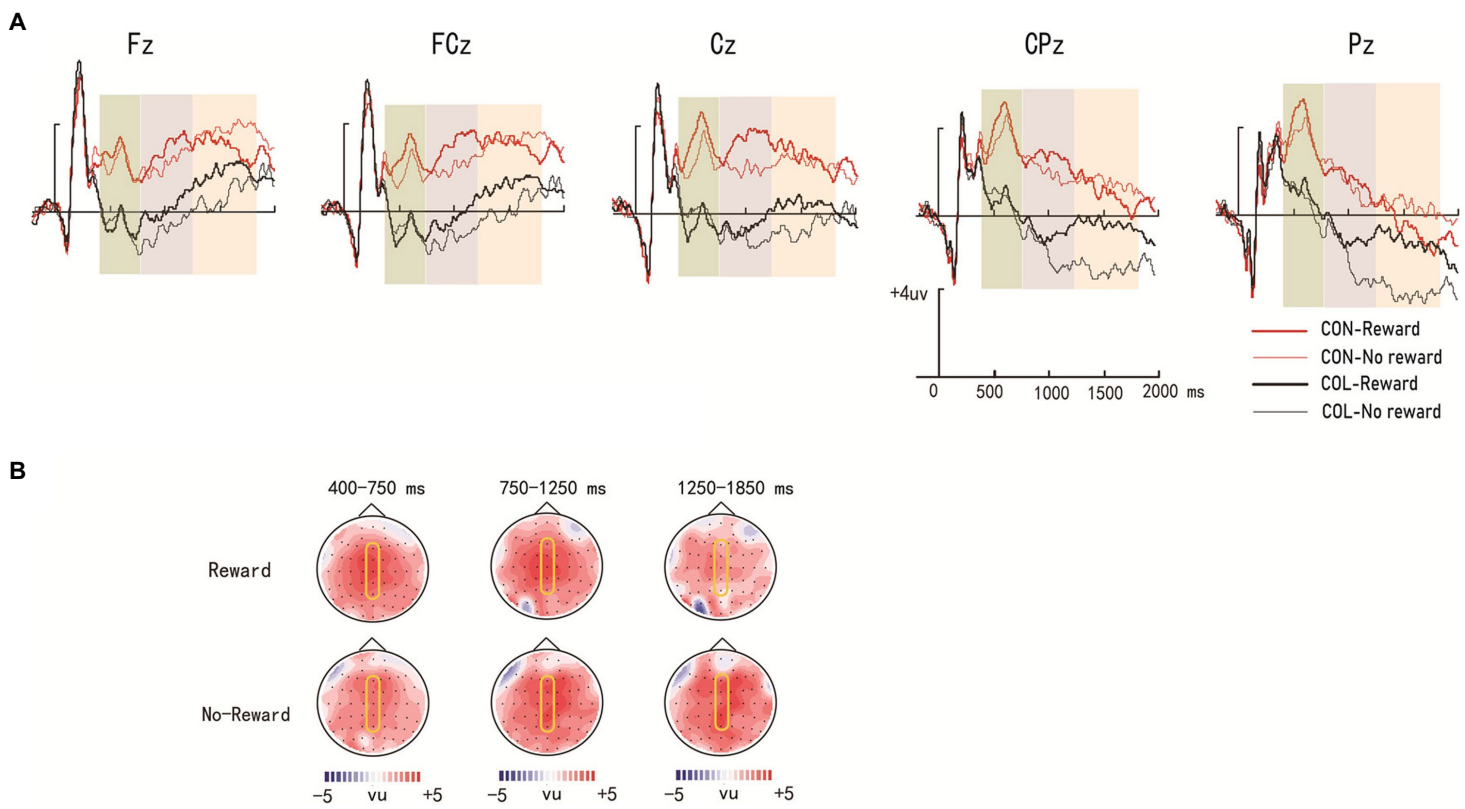
Amplitude distribution and topographic maps of event-related potentials (ERPs) during encoding. **(A)** Amplitude distribution of ERP measurements in relation to reward effects and encoding task effects. **(B)** Topographic maps of ERPs on the effect of encoding task on the object items encoded under reward and nonreward conditions.

**Figure 4:**
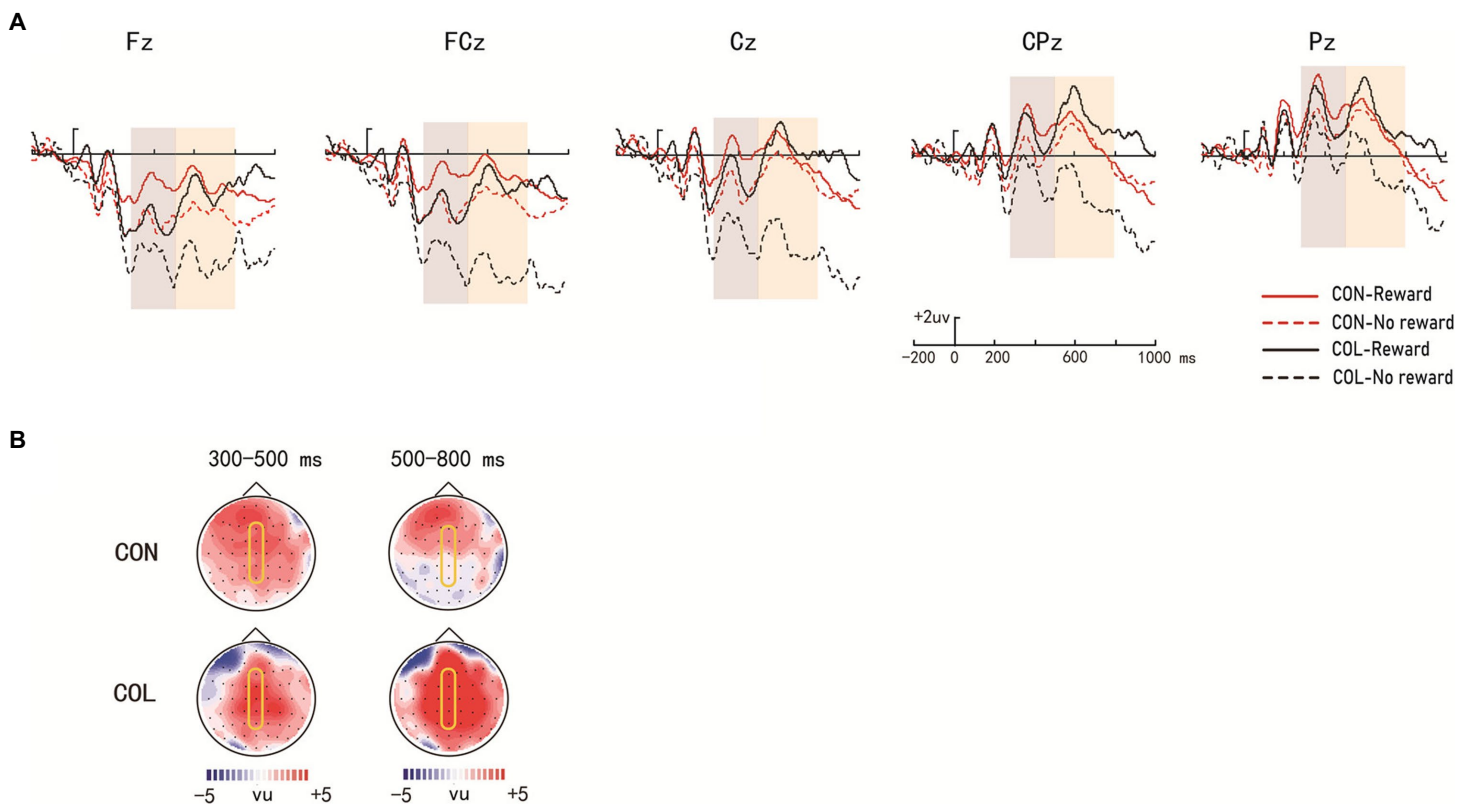
Amplitude distribution and topographic maps of ERP measurements of reward effects for intact items during retrieval. **(A)** Amplitude distribution of ERP measurements in relation to reward effects and encoding task effects. **(B)** Topographic maps of ERPs on reward effects in the items encoded through the two encoding tasks.

**Figure 5:**
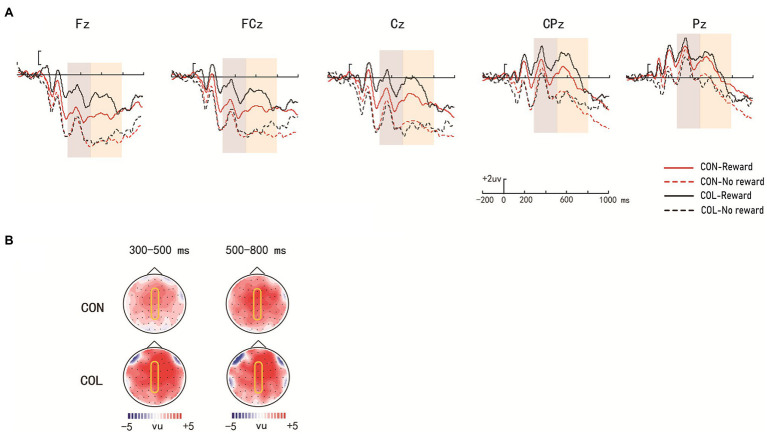
Amplitude distribution and topographic maps of ERP measurements of reward effects for rearranged items during retrieval. **(A)** Amplitude distribution of ERP measurements in relation to reward effects and encoding task effects. **(B)** Topographic maps of ERPs on reward effects in the items encoded through the two encoding tasks.

Figure 3 illustrates the ERP average amplitude distributions in the encoding phase. Figures 4, 5 illustrate the ERP average amplitude distributions in the test phase (Figure 4 illustrates the ERP average amplitude distributions for intact items; Figure 5 illustrates the ERP average amplitude distributions for rearranged items).

### Reward Effect and the Effect of the Encoding Task in the Encoding Phase

To analyze the effect of the encoding task and reward type, 2 (encoding task: congruity-judgment task vs. color-judgment task) × 2 (reward type: reward vs. nonreward) × 5 (electrode location: Fz, FCz, Cz, CPz, and Pz) repeated-measures ANOVAs were conducted on the average amplitudes of each time window.

#### Time Window of 400–750 ms

There were significant main effects of encoding task and electrode location [*F* _(1, 29)_ = 45.43, *p* < 0.001, $\eta_{p}^{2}$ = 0.61; *F*_(4, 26)_ = 10.99, *p* < 0.001, $\eta_{p}^{2}$ = 0.63], no significant main effect of reward type [*F*_(1, 29)_ = 1.68, *p* = 0.205, $\eta_{p}^{2}$ = 0.06], and a significant three-way interaction [*F*_(4, 26)_ = 3.48, *p* = 0.021, $\eta_{p}^{2}$ = 0.35]. Further simple effect analysis revealed that the average amplitudes in the congruity-judgment task were more positive than those in the color-judgment task (LPP) regardless of reward condition (*ps* < 0.05) at electrodes Fz, FCz, Cz, CPz, and Pz.

#### Time Window of 750–1,250 ms

There was a significant main effect of encoding task [*F*_(1, 29)_ = 38.26, *p* < 0.001, $\eta_{p}^{2}$ = 0.57] and a significant three-way interaction [*F*_(4, 26)_ = 3.15, *p* = 0.036, $\eta_{p}^{2}$ = 0.27], but no significant main effects of reward type and electrode location [*F*_(1, 29)_ = 3.48, *p* = 0.072, $\eta_{p}^{2}$ = 0.11; *F*_(4, 26)_ = 2.55, *p* = 0.063, $\eta_{p}^{2}$ = 0.28]. Further simple effect analysis indicated that the average amplitudes in the congruity-judgment task were more positive than those in the color-judgment task (LPP) regardless of reward condition (*ps* < 0.05) at electrodes Fz, FCz, Cz, CPz, and Pz; a significant reward effect was found only in the color-judgment task (*p* = 0.042) at electrode Pz.

#### Time Window of 1,250–1,850 ms

There was a significant main effect of the encoding task [*F*_(1, 29)_ = 18.25, *p* < 0.001, $\eta_{p}^{2}$ = 0.39], and electrode location [*F*_(4, 26)_ = 24.81, *p* < 0.001, $\eta_{p}^{2}$ = 0.79] and a significant three-way interaction [*F*_(4, 26)_ = 2.78, *p* = 0.045, $\eta_{p}^{2}$ = 0.26], but no significant main effect of reward type was [*F*_(1, 29)_ = 0.72, *p* = 0.404, $\eta_{p}^{2}$ = 0.02]. Further simple effect analysis showed that the average amplitudes in the congruity-judgment task were more positive than those in the color-judgment task (LPP) under the nonreward condition (*ps* < 0.05) at electrodes Fz, FCz, Cz, CPz, and Pz and under the reward condition (*ps* < 0.05) only at electrodes FCz and Cz; significant reward effects were found in the color-judgment task (*ps* < 0.05) at electrodes CPz and Pz.

### ERPs in the Test Phase

#### Reward Effect and the Effect of the Encoding Task in the Test Phase

To further analyze the effect of encoding task and reward type, 2 (encoding task: congruity-judgment task vs. color-judgment task) × 2 (reward type: reward vs. nonreward) × 5 (electrode location: Fz, FCz, Cz, CPz, and Pz) repeated-measures ANOVAs were conducted on the average amplitudes of each time window for intact and rearranged items.

##### Intact Items

###### Time Window of 300–500 ms

There were significant main effects of encoding task, reward type, and electrode location [*F*_(1, 29)_ = 12.71, *p* = 0.001, $\eta_{p}^{2}$ = 0.31; *F*_(1, 29)_ = 5.17, *p* = 0.031, $\eta_{p}^{2}$ = 0.16; *F*_(4, 26)_ = 21.50, *p* < 0.001, $\eta_{p}^{2}$ = 0.78] and a significant three-way interaction [*F*_(4, 26)_ = 16.96, *p* < 0.001, $\eta_{p}^{2}$ = 0.64]. Further simple effect analysis indicated that the reward effect at P300 was significant in the color-judgment task (*ps* = 0.041) at electrode FCz, and the average amplitudes in the congruity-judgment task were more positive than those in the color-judgment task under the nonreward condition (*ps* < 0.01) at electrodes FCz and Cz.

###### Time Window of 500–800 ms

There were significant main effects of encoding task, reward type, and electrode location [*F*_(1, 29)_ = 10.36, *p* = 0.032, $\eta_{p}^{2}$ = 0.11; *F*_(1, 29)_ = 11.23, *p* = 0.023, $\eta_{p}^{2}$ = 0.27; *F*_(4, 26)_ = 7.41, *p* < 0.001, $\eta_{p}^{2}$ = 0.54] and a significant three-way interaction [*F*_(4, 26)_ = 4.27, *p* = 0.009, $\eta_{p}^{2}$ = 0.41]. Further simple effect analysis revealed significant reward effects at LPP in the color-judgment task (*ps* < 0.05) at electrodes Fz, FCz, Cz, CPz, and Pz, and the average amplitudes in the congruity-judgment task were more positive than those in the color-judgment task under the nonreward condition (*ps* < 0.05) at electrodes Fz, FCz, and Cz.

##### Rearranged Items

###### Time Window of 300–500 ms

There were significant main effects of reward type and electrode location [*F*_(1, 29)_ = 5.62, *p* = 0.042, $\eta_{p}^{2}$ = 0.13; *F*_(4, 26)_ = 28.57, *p* < 0.001, $\eta_{p}^{2}$ = 0.82] and a significant three-way interaction [*F*_(4, 26)_ = 3.89, *p* = 0.046, $\eta_{p}^{2}$ = 0.29], but no significant main effect of encoding task [*F*_(1, 29)_ = 1.12, *p* = 0.298, $\eta_{p}^{2}$ = 0.04]. Further simple effect analysis indicated there were significant reward effects at P300 in the color-judgment task (*ps* < 0.05) at electrodes Fz and FCz, and the average amplitudes in the color-judgment task were more positive than those in the congruity-judgment task under reward conditions (*ps* < 0.05) at electrodes Fz, FCz, and Cz.

###### Time Window of 500–800 ms

There were significant main effects of reward type and electrode location [*F*_(1, 29)_ = 10.28, *p* = 0.035, $\eta_{p}^{2}$ = 0.15; *F*_(4, 26)_ = 14.91, *p* < 0.001, $\eta_{p}^{2}$ = 0.71] and a significant three-way interaction [*F*_(4, 26)_ = 5.21, *p* = 0.034, $\eta_{p}^{2}$ = 0.31], but no significant main effect of encoding task [*F*_(1, 29)_ = 0.63, *p* = 0.433, $\eta_{p}^{2}$ = 0.02]. Further simple effect analysis suggested that the reward effects at LPP were significant in the color-judgment task (*ps* < 0.05) at electrodes Fz, FCz, Cz, and CPz, and the average amplitudes in the color-judgment task were more positive than those in the congruity-judgment task under reward conditions (*ps* < 0.05) at electrodes Fz, FCz, Cz, and CPz.

#### Old/New (Intact/Rearranged) Effect

To analyze the old/new (i.e., intact/rearranged) effect, 2 (encoding task: congruity-judgment task vs. color-judgment task) × 2 (reward type: reward vs. nonreward) × 2 (item type: intact vs. rearranged) × 5 (electrode location: Fz, FCz, Cz, CPz, and Pz) repeated-measures ANOVAs were conducted on the average amplitudes of each time window.

##### Time Window of 300–500 ms

There was a significant main effect of the response [*F*_(1, 29)_ = 12.04, *p* = 0.002, $\eta_{p}^{2}$ = 0.30] and a significant three-way interaction [*F*_(4, 26)_ = 3.76, *p* = 0.016, $\eta_{p}^{2}$ = 0.38] among encoding task, response, and electrode location. Further simple effect analysis showed that the average amplitudes for the intact items were more positive than those for the rearranged items in the congruity-judgment task (*ps* < 0.05) at electrodes Fz, FCz, Cz, CPz, and Pz, supporting an FN400 old/new effect (peaks at approximately 480 ms poststimulus).

##### Time Window of 500–800 ms

There was a significant main effect of the response [*F*_(1, 29)_ = 20.65, *p* < 0.001, $\eta_{p}^{2}$ = 0.42], and the average amplitudes for the intact items were more positive than those for rearranged items regardless of task, indicating an LPC old/new effect.

## Discussion

The current study explored the effects and neural mechanisms of encoding tasks and rewards on between-domain inter-item associative recognition by using ERP techniques and the associative recognition paradigm. In the present study, we mainly explored relational retrieval. This study found that the reward differences of the Prs in the color-judgment task were greater than those in the congruity-judgment task and that the reward effects at P300 and LPP were greater for the color-judgment task.

During the encoding phase, the behavioral results showed that the congruity-judgment task had longer response times than the color-judgment task under the reward condition, and the rewarded items had a shorter response time in the color-judgment task than the nonrewarded items, which suggests that the encoding task and reward status had a mutual effect on the encoding process of associative memory. The ERP results further showed that there were more positive average amplitudes for items in the congruity-judgment task than for those in the color-judgment task at electrodes Fz, FCz, Cz, CPz, and Pz at 400–750 ms (LPP), 750–1,250 ms (LPP), and 1,250–1850 ms (LPP), both in reward and nonreward conditions, as we had expected. According to previous studies, LPP might reflect memory encoding; the average amplitudes of items with deeper processing strategies were more positive than those with shallower processing strategies (Marini et al., 2011). Their results revealed a greater investment of cognitive resources and deeper-level processing for the congruity-judgment task with high unitization (Moser et al., 2014; Shafir et al., 2015). In addition, the current ERP results further showed that a significant reward effect only in the color-judgment task at 750–1250 ms (LPP) and 1,250–1850 ms (LPP). This result might indicate that the reward effect in tasks with low unitization occurs during encoding.

During the test phase, the behavioral results showed that the reward differences in the Prs in the color-judgment task were greater than those in the congruity-judgment task. The behavioral results also showed that RTs were significantly shorter in the congruity-judgment task under the nonreward condition than those in the color-judgment task for the old/intact items. These results revealed that relational retrieval in the congruity-judgment task (Prs = 0.72) was relatively easier than that in the color-judgment task (Prs = 0.48). Previous studies have shown that the reward effect on cognitive task performance is highest when the cognitive control requirement is at a medium difficulty (Elward et al., 2015). We suggest that this may be because participants are better at remembering items with high unitization, and memory performance was less affected by reward. Shigemune et al. (2017) also found reward-related enhancement of memory only when memory retrieval was difficult. We predicted that the color-judgment task with low unitization would result in relatively more difficult retrieval (closer to the medium difficulty of 0.50), which could have caused the greater reward differences in Prs and wider ERP distribution of the reward effects in the color-judgment task compared to the congruity-judgment task. As shown in the ERP results, significant reward effects at P300 (300–500 ms) and LPP (500–800 ms) were found in the color-judgment task for the intact items; significant reward effects at P300 (300–500 ms) and LPP (500–800 ms) were also found in the color-judgment task for the rearranged items. According to previous ERP studies, P300 might reflect participants’ initial attention allocation to the stimulus, whereas LPP might reflect the significance of the stimulus (Foti and Hajcak, 2008; Hajcak et al., 2009). Therefore, these results, showing significant reward effects at P300 and LPP only in the color-judgment task with low unitization for both intact and rearranged items, indicate that more attention was given to the rewarded items in the low unitization task. In addition, the ERP results also showed that the reward effects at LPP (electrodes Fz, FCz, Cz, CPz, and Pz) were more widely distributed than the reward effects at P300 (electrodes Fz and FCz) in the color-judgment task, which might indicate that reward was more important in the later stage of relational retrieval. These results indicate that the connection of information with reward, relative to nonrewarded information, in the task with low unitization required a greater cognitive resource investment and greater motivation. In addition, reward was more important in the later stage of relational retrieval.

Previous ERP studies have shown that the FN400 old/new effect indicates familiarity, and the LPC old/new effect indicates recollection (Curran and Hancock, 2007; Addante et al., 2012). The unitization hypothesis posits that associative memory is also affected by familiarity when two items can be integrated into a unit (Yonelinas, 2002). Mayes et al. (2007) suggested that both intra-item associative recognition and within-domain inter-item associative recognition could be supported by familiarity, while between-domain inter-item associative recognition could only be supported by recollection. In this study, FN400 old/new effects (peaks at approximately 480 ms poststimulus) were found only in the congruity-judgment task, and LPC old/new effects were found in both the congruity-judgment and color-judgment tasks. Han et al. (2018) also found that unitization encoding was accompanied by enhanced recollection and familiarity. The results of the current study indicate that relational retrieval in the color-judgment task was influenced by recollection and that relational retrieval in the congruity-judgment task was influenced by both familiarity and recollection, which suggests that in the congruity-judgment task participants integrated words and images during encoding. We expected that between-domain inter-item associative recognition would be affected by familiarity when two items were linked together. However, familiarity plays an important role in between-domain inter-item associative memory at later points than in item memory.

Between-domain inter-item associative memory is the kind of memory that we use most often in everyday life, such as associating people’s names with their faces. Rewards work better at optimal retrieval difficulty of connected information. The present study also had limitations. We only compared the task with optimal retrieval difficulty and the task with easier retrieval difficulty; future research should further compare the differences between tasks that vary in retrieval difficulty with optimal retrieval difficulty tasks.

## Conclusion

In this ERP study, we investigated the effects and neural mechanisms of different unitization depths and reward anticipation sets in encoding in between-domain inter-item associative memory using ERPs. The behavioral results during encoding showed that the congruity-judgment task with high unitization had longer response times under the reward condition. The ERP results further showed that there were more positive average amplitudes for items in the congruity-judgment task (LPP). The associative memory retrieval behavioral results showed that the reward differences in the Prs in the color-judgment task were greater than those in the congruity-judgment task. The ERP results showed that significant reward effects at P300 and LPP were found in the color-judgment task both for intact and rearranged items and that the reward effects at LPP were distributed more widely than the reward effects at P300 in the color-judgment task. In addition, the ERP results also showed that between-domain inter-item associative recognition was affected by familiarity when two items were linked together. Overall, reward provided a greater boost when retrieving associative memory of low unitized items.

## Data Availability Statement

The original contributions presented in the study are included in the article/supplementary material, further inquiries can be directed to the corresponding author.

## Author Contributions

CY supervised the project. CY and QD designed the experiment, wrote the main manuscript text, and prepared the Figures 1–5 and Table 1. QD collected and analyzed the experimental data. CY, QD, MW, and JZ reviewed the manuscript. All authors contributed to the article and approved the submitted version.

## Funding

This research was supported by the philosophy and social science research project (2018BJY024 and 2020BJY028) in Henan Province and the Humanities and Social Science Project (2019-ZZJH-526) of Henan Province Office of Education in China.

## Conflict of Interest

The authors declare that the research was conducted in the absence of any commercial or financial relationships that could be construed as a potential conflict of interest.

## Publisher’s Note

All claims expressed in this article are solely those of the authors and do not necessarily represent those of their affiliated organizations, or those of the publisher, the editors and the reviewers. Any product that may be evaluated in this article, or claim that may be made by its manufacturer, is not guaranteed or endorsed by the publisher.
